# Rest–activity rhythm associated with depressive symptom severity and attention among patients with major depressive disorder: a 12-month follow-up study

**DOI:** 10.3389/fpsyt.2023.1214143

**Published:** 2023-08-10

**Authors:** Hang-Ju Yang, Wan-Ju Cheng, Mi-Chun Hsiao, Sheng-Che Huang, Tomohide Kubo, Liang-Wen Hang, Wei-Sheng Lee

**Affiliations:** ^1^Department of Emergency Medicine, Jen Ai Hospital Dali Branch, Taichung, Taiwan; ^2^Department of Psychiatry, China Medical University Hospital, Taichung, Taiwan; ^3^National Center for Geriatrics and Welfare Research, National Health Research Institutes, Miaoli, Taiwan; ^4^Center for Drug Abuse and Addiction, China Medical University Hospital, China Medical University, Taichung, Taiwan; ^5^Department of Public Health, China Medical University, Taichung, Taiwan; ^6^National Institute of Occupational Safety and Health, Kawasaki, Japan; ^7^Department of Pulmonary and Critical Care Medicine, Sleep Medicine Center, China Medical University Hospital, Taichung, Taiwan; ^8^School of Nursing and Graduate Institute of Nursing, China Medical University, Taichung, Taiwan

**Keywords:** sleep, chronotype, mood disorder, cognition, rhythm

## Abstract

**Introduction:**

Patients with depressive disorder demonstrate rest–activity rhythm disturbances and cognitive function impairment. This study examined the association of individual rest–activity rhythm changes over time with mood symptoms and attention.

**Methods:**

We recruited 15 adult outpatients with a diagnosis of major depressive disorder from a single medical center and observed them for 12 months. Weekly rest–activity parameters, including rhythm characteristics generated from nonparametric circadian rhythm analysis, were retrieved from actigraphy data. Attention was evaluated weekly with a smartphone-based psychomotor vigilance test upon awakening. Depressive symptom severity was evaluated using the Beck Depression Inventory (BDI) fortnightly. The association of rest–activity parameters with BDI score and attention was examined using generalized linear mixed regression. A fixed-effects analysis was used to examine the association between rest–activity parameters and depressive episodes.

**Results:**

An advanced bedtime and most active continuous 10 h starting time were associated with depressive symptom severity but also associated with higher vigilance test performance. A longer sleep duration, mainly due to an earlier bedtime, was associated with depressive symptom severity. Compared to remission, sleep duration was 27.8 min longer during depressive episodes, and bed time was 24 min earlier. A shorter sleep duration and increased activity during sleep were associated with poorer attention.

**Discussion:**

Rest–activity rhythms change with mood symptoms among patients with depressive disorder. The circadian rhythms of rest–activity among patients with depressive disorder should be distinguished during various mood states in future studies.

## 1. Introduction

Nearly all patients with depressive disorder have significant circadian rhythm disturbance and sleep disruptions, and several physiological mechanisms (e.g., monoamine transmission, HPA axis function, and immune function) have been proposed (1). The relationship between circadian rhythm disturbance and major depressive disorder is bidirectional (2), meaning that circadian rhythm disruptions may be either a risk factor or a result of mental health problems. Additionally, interventions that are based on circadian rhythms have shown encouraging clinical results for improving mood symptoms (3, 4).

The central regulator of circadian rhythm that drives REM sleep, temperature, melatonin, and cortisol is hypothesized to be abnormally advanced in time among patients with depression (5). Studies have reported that biological markers of circadian rhythm, as measured by core temperature, indicated more advanced rhythms (6) and lower variability (7) during depression compared with during remission (6), but the biological markers did not differ from those of healthy people (8, 9). These findings suggest that circadian rhythm change may be a state rather than a trait of depressive episodes (2).

Rest–activity patterns may reflect the central rhythm of biological markers while also provide feedback and modify central circadian rhythms (10). Measuring circadian rhythms, such as through core body temperature, dim-light melatonin onset (DLMO), or cortisol level, is costly, and continually observing human patients is difficult (11). Actigraphy is used to continually monitor physical activities and has been used to characterize rest–activity rhythms (12). Actigraphy has been used to monitor activity levels and sleep patterns among patients with depressive disorders (13–15). Studies using actigraphy have observed that patients with depressive disorder engaged in less daytime activity compared with healthy individuals (16). However, few studies examined differences in rest–activity rhythms between patients with depressive disorder and healthy people. A study using a 1-week actigraphy reported lower mean activity and dampened amplitude among patients with depressive disorder compared with healthy individuals (17, 18). Advanced phase estimated by actigraphy was associated with depression in adolescent boys (19). By conducting a nonparametric analysis of 1 week of actigraphy data, a population-based study observed that a reduction in relative amplitude was associated with an increased risk of lifetime major depressive disorder and lifetime bipolar disorder (20). In these studies, rest–activity patterns were measured once and the episodic nature of mood disorders was not accounted for. Studies examining changes in the rest-activity rhythm across different mood states among patients with mood disorders have been limited. One study indicated an advanced circadian phase during manic episodes, which returned to normal with treatment (21).

Cognitive impairment is one of the major symptoms of depression, and sleep disturbance exacerbates cognitive dysfunction through serotonergic signaling (22). Additionally, chronic sleep deprivation can lead to depression that is characterized by impaired brain-derived neurotrophic factor expression and low diurnal variability in the brain. Furthermore, sleep deprivation was associated with impaired memory and cognitive functions (23). However, these observations have been restricted to animal studies, and the association of rest–activity rhythm with depression and cognitive function have rarely been studied.

Other studies investigating the association between rest–activity rhythm and depressive symptoms monitored sleep and activities for less than 1 month (16) and assumed a fixed rest–activity pattern over time. However, the episodic course of major depressive disorder limits the validity of short-term actigraphy monitoring for determining rest–activity rhythms. Therefore, whether rest–activity patterns persist throughout the disease course or change with mood symptoms is unclear due to a lack of longitudinal studies (24). In this study, we examined the intra-individual association of rest–activity rhythm with mood symptoms and attention over time among patients with depressive disorder. We hypothesized that the severity of mood symptoms is associated with an advanced rest-activity rhythm.

## 2. Materials and methods

### 2.1. Study participants

We recruited 15 adult outpatients with a diagnosis of major depressive disorder (ICD-10: F32-F33) from a single medical center. The participants were interviewed by board-certified psychiatrists who confirmed their diagnoses according to the *Diagnostic and Statistical Manual of Mental Disorders, Fifth Edition*. The participants were referred to the research team and provided informed consents. Age, sex, past disease history, habitual sleep time, and working time were interviewed by the research team and also retrieved from medical charts. The exclusion criteria were having (1) comorbidities of schizophrenia or schizoaffective disorder or substance use disorder, except for tobacco use disorder; (2) a confirmed diagnosis of narcolepsy, hypersomnia, or severe obstructive sleep apnea; and (3) nonstandard working hours (i.e., working more than 3 h beyond 06:00 to 18:00). This study was approved by the Institutional Review Board of China Medical University Hospital [CMUH 109-REC1-186].

### 2.2. Study design

Each patient was observed for up to 1 year after being recruited to the study between February and December 2021. Mood symptoms, sleep quality ratings, and rest–activity parameters retrieved from wrist actigraphy were collected during the following 12 months (Supplementary Figure 1). The patients were instructed to wear an actigraphy (MotionWatch 8; CamNTech, Cambridge, United Kingdom) on their non-dominant wrists continuously after recruitment, and electronic data were collected monthly by research assistants. The participants were contacted by the research team every 2 weeks after recruitment (baseline), and were considered lost to follow-up if they refused further assessments or could not be contacted for more than 4 weeks. During the one-year follow-up period, patients were required to maintain their psychiatric medications, including antidepressants, antipsychotics, or sedatives, and dosages could not be adjusted by more than 50%. The follow-up would terminate if a significant change in medication treatment was decided to be the best course of action by the patient’s psychiatrist after clinical assessment.

### 2.3. Measurements for rest–activity pattern and sleep quality

Actigraphy data were recorded in 60-s epochs and analyzed in 1-week intervals. A button on the actigraphy was pressed by the participants to assist sleep scoring, which signified bedtime, get-up time, take off and put on actigraphy. We retrieved weekly averaged bedtime, fall-asleep time, wake-up time, get-up time, sleep duration, sleep onset latency, and duration of wake after sleep onset. The rhythm parameters by week were interdaily stability, least active continuous 5 h (L5) activity counts and onset time, most active continuous 10 h (M10) activity counts and onset time, relative amplitude, and fitted peak time of activity (25). Data were generated using a nonparametric circadian rhythm analysis and cosinor analysis in MotionWare 1.2.28 (CamNTech, Cambridge, United Kingdom). Interdaily stability ranged from 0 to 1, with higher values indicating higher invariability of the 24-h rhythm. Relative amplitude is the difference between M10 and L5 divided by the sum of M10 and L5 activity. Relative amplitude ranged from 0 to 1, with higher values indicating a clearer distinction between activity levels during the most and least active periods of the day. Subjective sleep quality was assessed using the Pittsburgh Sleep Quality Index (PSQI) (26) once every week.

### 2.4. Measurements for mood symptoms and attention

The primary outcome was depressive symptom severity, which was assessed using the self-administered Beck Depression Inventory (BDI-II) fortnightly. A BDI score ≥ 19 was used to identify a depressive episode (27). Attention was evaluated weekly by using a smartphone-based 5-min psychomotor vigilance test (PVT) upon awakening (28). The participants received written instructions in Mandarin, guiding them on how to use the test in Japanese, and they underwent one test trial during the study introduction to ensure appropriate usage before the formal test. The original settings were retained, with a random interstimulus interval ranging from 2 to 10 s. The number of lapses (i.e., a response of longer than 0.5 s), reaction time (in seconds), and error number were counted during each 5-min test.

### 2.5. Measurements for circadian rhythm marker

Salivary assessment of DLMO has been used to identify circadian rhythms and has demonstrated high accuracy outside of laboratory settings (29). The patients provided saliva samples once at home during remission (determined according to BDI score < 19). Saliva samples were collected in sterile 50-mL Salivette tubes every 30 min between 5 h before and 1 h after the usual bedtime. Instructions were given regarding dim light condition, mouth cleaning and diet control (30, 31). All saliva samples were frozen at −20°C until being assayed. After thawing, a centrifugation at 2,000× g for 10 min at room temperature was performed to remove particulate material before employing Melatonin Direct Saliva ELISA (LDN Labor Diagnostika, Nordhorn, Germany) to obtain the melatonin levels according to the manufacturer’s instructions. The melatonin levels were examined in duplicate, and the mean values of the duplicates were used for calculations. The limit of quantitation (LoQ) of the kit was 0.854 pg/mL. DLMO was determined according to the two standard deviation (SD) threshold method, which uses the average of the first three melatonin data points plus two SDs of the same three points (32). Six patients failed to collect adequate saliva samples due to mouth dryness or being unable to comply with the saliva collection schedule. The DLMO was calculated for the other nine patients.

### 2.6. Statistical analysis

We examined the association of rest–activity parameters with BDI score and attention performance in PVT tests by using a generalized linear mixed regression. Age, sex, and DLMO were between-subject variables, and rest–activity parameters were within-subject variables. In the regression models, DLMO was dichotomized into early and late groups by median, and a dummy variable was created for missing data. L5 and M10 activity were log-transformed in the regression models because the distributions of both were skewed. A fixed-effects analysis was used to examine the association between rest–activity parameters and depressive episodes (defined by a BDI score ≥ 19). The differences in rest–activity parameters between depressive episodes and remission were calculated. SAS 9.4 (SAS Institute, Cary, NC, United States) was used for all analyses.

## 3. Results

Table 1 presents the baseline characteristics of the patients. Seven of the 15 patients completed 52 weeks of follow-up, eight patients were lost of follow-up prematurely. None of the patients dropped out due to medication adjustments. The average follow-up period was 42.1 weeks (SD = 13.8). The patients exhibited a moderate degree of depressive symptoms at baseline (BDI = 24.9) and had an average PSQI score of 11.2 (SD = 3.5). The average DLMO for the nine patients who completed the sample collection was at 21:47. Baseline rest–activity parameters indicated an average bedtime of 23:36 and get-up time of 07:48. The average total sleep time was 6.5 h.

**Table 1:** Baseline characteristics of patients (*N* = 15).

	Mean or *N*	Standard deviation or %
Age	43.1	15.7
Sex (female *N*, %)	13	83.3%
Depressive disorder history (years)	5.9	4.8
Follow-up weeks	42.1	13.8
**Medications (*N* and %)**		
Antidepressants	14	93.3%
Antipsychotics	7	46.7%
Benzodiazepines and Z-drugs	10	60%
**Symptom severity**		
BDI score	24.9	13.4
PSQI score	11.2	3.5
Dim light melatonin onset (*N* = 9)	21:47	02:49
**Psychomotor vigilance test**		
Reaction time (ms)	714.3	330.0
Lapse number	13.0	13.0
Error number	1.3	2.0
**Rest-activity parameters**		
Bedtime	23:36	1:18
Fall-asleep time	0:18	1:25
Wake-up time	7:46	1:52
Get-up time	7:48	1:54
Midsleep time	3:58	1:23
Total sleep time (mins)	391.5	92.6
Sleep onset latency (mins)	15.2	15.9
Wake after sleep onset (mins)	74.0	18.9
Interdaily stability	0.49	0.12
Fitted cosine peak	15:12	1:36
Least 5 h starting time	4:42	5:00
Least 5 h activity counts (h^−1^)	266.2	172.4
Most 10 h starting time	10:12	2:00
Most 10 h activity counts (h^−1^)	1533.3	793.8
Relative amplitude	0.82	0.11

BDI, Beck depression inventory; PSQI, Pittsburgh sleep quality index.

**Figure 1:**
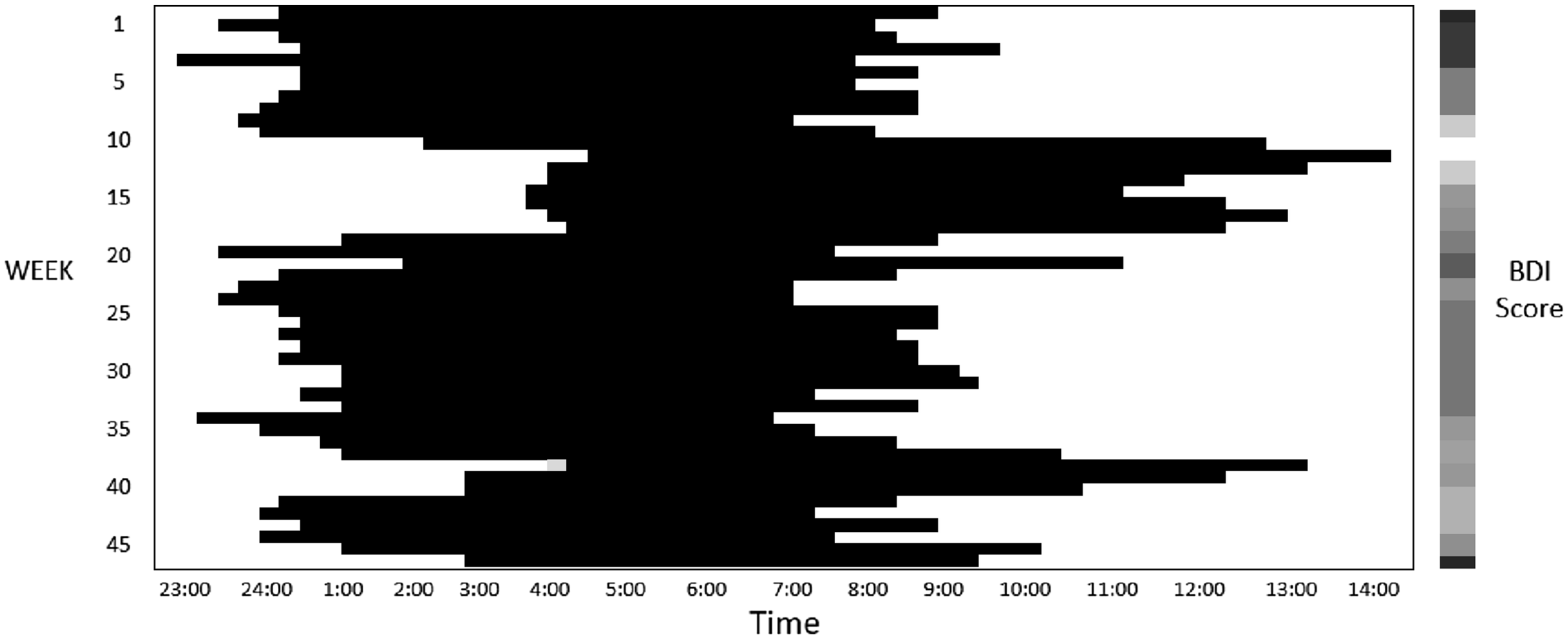
This figure presents a sample sleep pattern (where dark bars represent sleep) derived from actigraphy recordings for a study participant who was monitored for a duration of 47 weeks. The participant’s Beck Depression Inventory (BDI) score, assessed fortnightly, are depicted through a heatmap, with darker colors indicating higher score. This particular participant demonstrated BDI score ranging between 12 and 37 throughout the course of the study.

In the generalized linear mixed regression, we observed that bedtime (β = −1.89, *p* < 0.001) and M10 starting time (β = −0.58, *p* = 0.02) were negatively associated with depressive symptom score (Table 2). Hence, an earlier bedtime and earlier starting hour of daytime activity were associated with worse depressive symptoms. Figure 1 shows an example of a sleep pattern for one study participant during the follow-up period. By contrast, a later bedtime and M10 starting time were associated with more lapses in PVT. Subjective poor sleep quality (β = 1.61, *p* < 0.001) and longer duration of being wake after sleep onset (β = 0.08, *p* = 0.001) were both associated with depressive symptoms. A shorter total sleep time, lower relative amplitude, and higher L5 activity were associated with worse PVT performance. We further added the BDI score in the regression models to examine the association between the rest–wake parameters and PVT performance, and the results were similar to those of the nonadjusted models (Supplementary Table 1).

**Table 2:** Association between actigraphy-derived rest–activity parameters, mood symptoms, and psychomotor vigilance test results (*N* = 15).

	BDI score		Psychomotor vigilance test					
			Reaction time (ms)		Lapse number		Error number	
	Estimate (β)	*p*	Estimate (β)	*p*	Estimate (β)	*p*	Estimate (β)	*p*
Bedtime	−1.89	<0.001	29.65	0.30	1.15	<0.001	0.12	0.69
Fall-asleep time	−2.00	<0.001	37.19	0.19	1.15	<0.001	0.13	0.68
Wake-up time	0.14	0.75	−45.10	0.08	−0.07	0.78	0.37	0.17
Get-up time	0.21	0.65	−45.16	0.08	−0.04	0.87	0.33	0.22
Midsleep time	−0.95	0.09	−11.30	0.71	0.62	0.04	0.35	0.29
Total sleep time	0.02	0.01	−1.53	0.003	−0.02	<0.001	0.003	0.56
Sleep onset latency	−0.02	0.65	3.65	0.15	0.02	0.53	0.02	0.42
Wake after sleep onset	0.08	0.001	−1.03	0.33	−0.02	0.12	0.01	0.41
Interdaily stability	−0.81	0.83	−66.28	0.77	0.60	0.78	1.38	0.56
Cosine fitted peak time	−0.55	0.07	−30.0	0.10	0.81	<0.001	0.30	0.12
Least 5 h starting time	−0.21	0.55	−23.4	0.22	0.25	0.18	0.19	0.35
Least 5 h activity counts	−2.06	0.06	158.4	0.01	1.41	0.01	−0.14	0.82
Most 10 h starting time	−0.58	0.02	−1.34	0.92	0.58	<0.001	0.12	0.42
Most 10 h activity counts	−1.88	0.23	55.22	0.56	−0.32	0.73	−0.99	0.34
Relative amplitude	2.40	0.68	−668.2	0.04	−9.29	0.003	−2.26	0.51
PSQI score	1.61	<0.001	−6.93	0.53	−0.06	0.57	0.03	0.77

PSQI, Pittsburgh sleep quality index.

A total of nine patients had depressive episodes according to their BDI score during follow-up. Compared with during remission, the patients went to bed 24 min earlier and had a slightly longer total sleep time (28.6 min) during depressive episodes (Table 3). The M10 starting time was 12 min earlier during depressive episodes. Subjective sleep quality (i.e., the PSQI score) was worse, and wake after sleep onset was 10 min longer during depressive episodes. The average L5 activity was lower during depressive episodes compared with during remission.

**Table 3:** Mean and standard deviation (SD) in rest–activity parameters and attention performance between depressive episodes and remission.

	Depressive episodes		Remission		Odds ratio (95% CI)	*p*-value
	Mean	SD	Mean	SD		
**Rest-activity parameters**						
Bedtime	23:34	0:55	23:58	1:18	0.59 (0.37, 0.94)	0.03
Fall-asleep time	23:47	056	0:14	1:12	0.56 (0.35, 0.89)	0.02
Wake-up time	8:07	1:02	7:59	1:46	1.01 (0.67,1.52)	0.98
Get-up time	8:16	0:59	8:09	1:41	0.97 (0.64, 1.49)	0.91
Midsleep time	3:57	0:49	4:07	1:23	0.71 (0.44, 1.16)	0.18
Total sleep time (mins)	410.6	83.0	382.8	78.0	1.01 (1.00, 1.02)	0.04
Sleep onset latency (mins)	12.5	11.2	15.5	14.9	0.98 (0.94, 1.02)	0.33
Wake after sleep onset (mins)	91.3	48.7	81.3	41.1	1.03 (1.00, 1.05)	0.03
Interdaily stability	0.49	0.11	0.53	0.09	0.97 (0.94, 1.01)	0.13
Fitted cosine peak	15:53	0:22	15:53	1:27	0.82 (0.64, 1.06)	0.13
Least 5 h starting time	1:01	1:08	1:08	1:39	0.86 (0.63, 1.16)	0.38
Least 5 h activity counts (h^−1^)	197.5	109.7	233.8	122.4	0.28 (0.09, 0.84)	0.047
Most 10 h starting time	10:47	0:53	10:59	2:00	0.81 (0.65, 1.00)	0.06
Most 10 h activity counts (h^−1^)	1563.5	986.3	1655.0	872.9	0.30 (0.06, 1.53)	0.13
Relative amplitude	0.85	0.08	0.86	0.07	1.02 (0.96, 1.09)	0.67
PSQI score	11.9	4.8	8.5	2.9	1.58 (1.27,1.97)	<0.001
**Psychomotor vigilance test**						
Reaction time (ms)	825.4	740.4	605.4	232.2	1.20 (0.34, 4.25)	0.45
Lapse number	15.7	12.4	15.8	11.2	0.96 (0.91, 1.02)	0.27
Error number	7.2	16.3	10.0	23.7	0.90 (0.79, 1.04)	0.23

Right column shows odds ratio and 95% confidence interval (CI) for depressive episodes in fixed-effects analysis (*N* = 9). PSQI, Pittsburgh sleep quality index.

## 4. Discussion

In this longitudinal study, we observed a differential association of rest–activity rhythm with the severity of depressive symptoms and attention. An advanced rest–activity rhythm was associated with more severe depressive symptoms but better attention performance. A longer sleep time, mainly due to an earlier bedtime, was associated with depressive symptom severity, whereas a shorter sleep time and more activity during sleep were associated with worse attention.

Our finding that patients slept earlier during periods of worsened depressive symptoms may reflect advanced biological circadian rhythms. The DLMO among patients during nondepressive episodes was 21:47, which is slightly earlier than that in patients with bipolar disorder or healthy adults (30, 33) and similar to patients with depressive disorder (8). When the patients slept earlier at 23:34 during depressive episodes compared with 23:58 during remission, the DLMO–bedtime phase angle decreased. However, we did not measure the DLMO during depressive episodes, and the actual phase angle change cannot be determined. Clinical trials have indicated that chronotherapy that advances the sleep schedule among patients with major depressive disorder and evening chronotypes increased the remission rate (34–36). A study that treated a group of younger patients with unipolar depression indicated that an earlier sleep onset and shortened phase angle were associated with symptom improvement (36). Based on our observations, we suggest that a decreased phase angle was probably a result of advanced circadian rhythm during depressive episodes. In other words, patients with depressive disorders may tend to sleep earlier as a form of self-treatment.

In this study, the increased total sleep time during heightened depressive symptoms was mainly due to an earlier bedtime. A longer duration of being wake after sleep onset during heightened depressive symptoms led to worse sleep quality, as subjectively assessed using the PSQI. However, we observed a reduction in L5 activities during depressive episodes compared with during remission; this observation is inconsistent with findings from a longitudinal study that nighttime activity decreased after treatment for depressive disorder (16). The increase in L5 activity is likely a result of irregular rest–activity rhythms. Because L5 activity was generated by averaging 7 days of data in 24-h periods, the increase in L5 activity may not reflect activity during sleep time but instead activity during non-sleep period at night. Therefore, L5 and M10 activity should be interpreted in individuals with high interdaily stability in their activities. In our study, the interdaily stability was 0.49, which suggests low rest–activity regularity.

A short total sleep time, low relative amplitude, and increased L5 activity were associated with poorer attention. Sleep deprivation can worsen vigilant attention (37). In the present study, a delayed rest–activity rhythm (bedtime, midsleep time, M10 starting time, and cosine-fitted peak time) was associated with worse PVT performance, even after controlling for BDI score and DLMO. Studies have consistently shown that attention has circadian variations (38), and cognitive performance is maintained throughout the day due to opposing circadian and homeostatic processes (39). Because the PVT in the present study was performed when patients woke up, we speculate that on the weeks when patients experienced a delayed rest–activity rhythm, the optimal time for attention performance was delayed. Because the get-up time did not change during the follow-up period, the patients may have performed suboptimally upon awakening. To date, the synchrony effect that results from the interaction between chronotype and time-of-day has been inconsistently reported (40–42). More studies are necessary to understand the effects of the time of sleep, biological circadian rhythm, and homeostatic process on cognitive function among depressive patients.

This study has some strength. We used actigraphy to monitor rest–activity patterns over a long period, and an intra-individual comparison was applied so that biases originating from unobserved individual time-invariant characteristics (e.g., personality and genetic vulnerability) were eliminated. However, this study has some limitations. First, we did not follow up on the circadian rhythms of biological markers over time. DLMO may change with mood symptoms, and we were unable to determine the effect of DLMO and rest–activity rhythm on mood and attention. Furthermore, the home DLMO saliva test may be confounded by inadequate light exposure control. Second, the sample size was small. However, a long follow-up period was used, and data collected from multiple time-points provided adequate statistical power. Third, the patients were not drug-naïve, and their rest–activity patterns may have been influenced by medication. Nevertheless, an intra-individual comparison could minimize the effect of medication. Fourth, mood episodes were defined by BDI score, which was self-evaluated. The lack of clinical objective assessment may have led to bias in mood episode identification.

In conclusion, the rest–activity patterns of patients with depressive disorder are related with mood symptoms. An advanced rest–activity rhythm during depressive episodes is probably associated with an advanced circadian rhythm of biological markers, which is observed in other studies. Therefore, we suggest that future studies distinguish the rest–activity rhythms during depressive episodes and remission. Additionally, a delayed rest–activity rhythm is associated with poorer attention performance, regardless of the severity of depressive symptoms. To achieve optimal function, we suggest patients with depressive disorder avoid delayed bedtime.

## Data availability statement

The raw data supporting the conclusions of this article will be made available by the authors, without undue reservation.

## Ethics statement

The studies involving human participants were reviewed and approved by Institutional Review Board of China Medical University Hospital. The patients/participants provided their written informed consent to participate in this study.

## Author contributions

W-JC contributed to the conception and design of the study and drafted the manuscript. S-CH collected patient data. H-JY and W-SL performed the analysis of data and interpretation of results. TK provided critical technique support for vigilance test design and result interpretation. M-CH performed the saliva ELISA tests. L-WH provided critical comments on study design and result interpretation. All authors contributed to the article and approved the submitted version.

## Funding

This work was supported by the National Science and Technology Council, Taiwan grant number MOST 110-2314-B-039-022 to W-JC. The funders have no role in study design; in the collection, analysis and interpretation of data; in the writing of the report; and in the decision to submit the article for publication.

## Conflict of interest

The authors declare that the research was conducted in the absence of any commercial or financial relationships that could be construed as a potential conflict of interest.

## Publisher’s note

All claims expressed in this article are solely those of the authors and do not necessarily represent those of their affiliated organizations, or those of the publisher, the editors and the reviewers. Any product that may be evaluated in this article, or claim that may be made by its manufacturer, is not guaranteed or endorsed by the publisher.
