# Supplementary material for: Rest–activity rhythm associated with depressive symptom severity and attention among patients with major depressive disorder: a 12-month follow-up study
Source: Front Psychiatry. 2023 Aug 10;14:1214143. doi: 10.3389/fpsyt.2023.1214143 (PMC10469591; doi:10.3389/fpsyt.2023.1214143)
Supplement: Supplementary file 1 [file Data_Sheet_1.docx]

Supplementary Figure 1. Timeline of assessments in the study. BDI = Beck Depression Index; DLMO = dim light melatonin onset; PSQI = Pittsburgh sleep quality index; PVT = psychomotor vigilance test


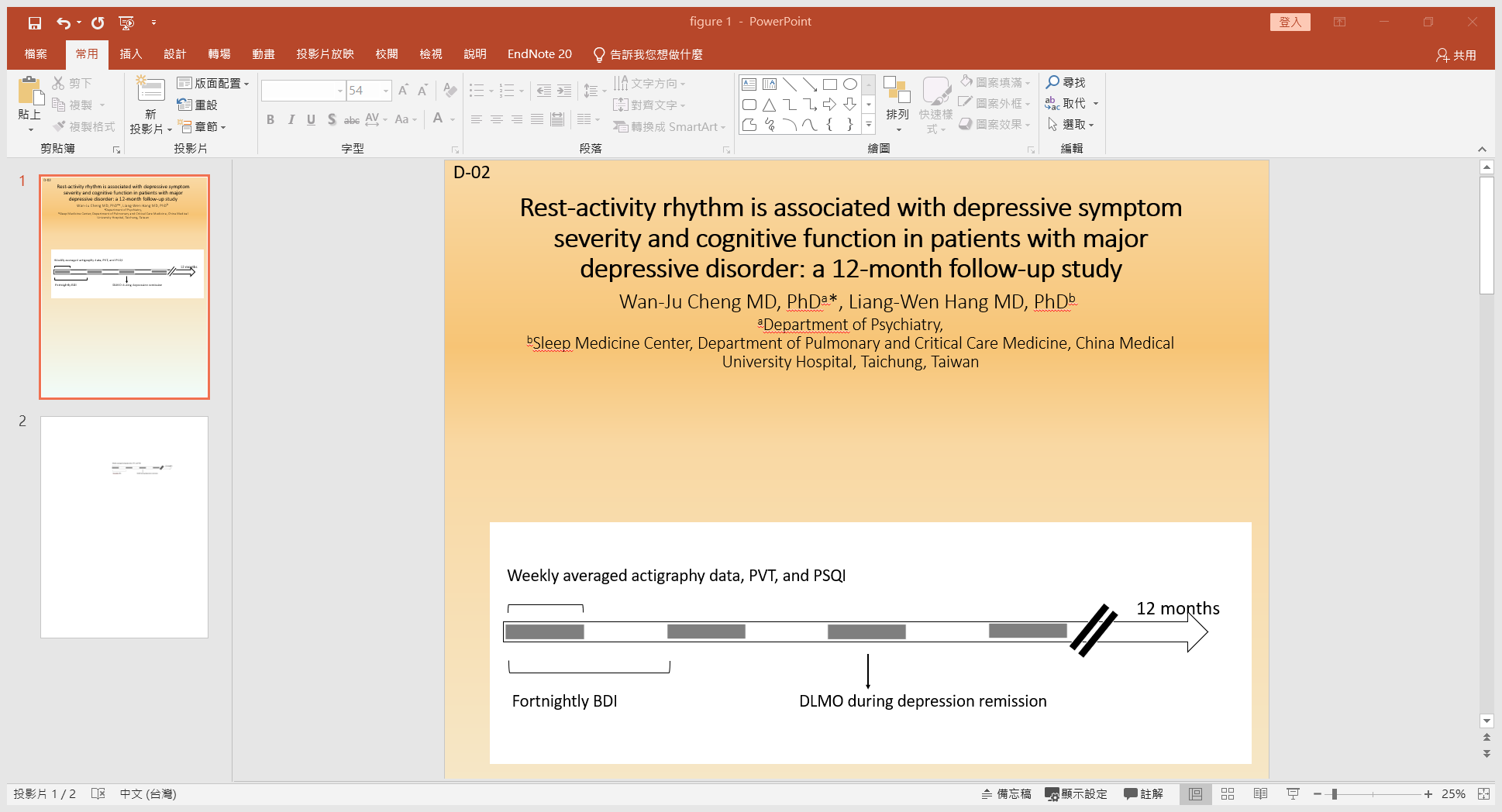


Supplementary Table 1. Association between actigraphy-derived rest–activity parameters and attention, adjusted for age, sex, dim-light melatonin onset, and Beck Depression Inventory score (*N* = 15).

|  | Reaction time (msec) | | Lapse number | | Error number | |
| --- | --- | --- | --- | --- | --- | --- |
|  | Estimate (β) | p | Estimate (β) | p | Estimate (β) | p |
| Bedtime | 2.12 | 0.96 | **1.15** | **0.07** | 0.02 | 0.97 |
| Fall-asleep time | 13.5 | 0.75 | **1.22** | **0.004** | -0.04 | 0.95 |
| Wake-up time | -48.7 | 0.16 | -0.19 | 0.59 | 0.01 | 0.98 |
| Get-up time | -47.5 | 0.17 | -0.20 | 0.57 | -0.09 | 0.84 |
| Midsleep time | -30.9 | 0.47 | 0.50 | 0.26 | -0.01 | 0.98 |
| Total sleep time | **-0.15** | **0.03** | **-0.02** | **0.002** | -0.01 | 0.46 |
| Sleep onset latency | 4.92 | 0.14 | 0.04 | 0.22 | 0.02 | 0.69 |
| Wake after sleep onset | 0.05 | 0.98 | -0.02 | 0.22 | 0.03 | 0.24 |
| Interdaily stability | 264.6 | 0.34 | 0.66 | 0.82 | 0.39 | 0.91 |
| Cosine fitted peak time | -33.4 | 0.16 | **0.74** | **0.002** | 0.08 | 0.80 |
| Least 5 hours starting time | -29.2 | 0.26 | 0.07 | 0.80 | 0.36 | 0.27 |
| Least 5 hours activity counts | **197.5** | **0.02** | 1.11 | 0.19 | 1.29 | 0.23 |
| Most 10 hours starting time | -17.1 | 0.37 | **0.52** | **0.006** | -0.09 | 0.69 |
| Most 10 hours activity counts | 96.6 | 0.41 | -0.94 | 0.45 | -0.70 | 0.66 |
| Relative amplitude | -413.8 | 0.35 | **-9.6** | **0.03** | -7.90 | 0.16 |
| PSQI score | -16.7 | 0.30 | -0.03 | 0.89 | 0.07 | 0.76 |
